# Evaluation of different methods for *in vitro* susceptibility testing of colistin in carbapenem resistant Gram-negative bacilli

**DOI:** 10.1099/acmi.0.000595.v3

**Published:** 2023-10-16

**Authors:** Bidyutprava Rout, Sumesh Kumar Dash, Kundan kumar Sahu, Birasen Behera, Ira Praharaj, Sarita Otta

**Affiliations:** ^1^​ Department of Microbiology, IMS and SUM Hospital, SOA University, Kalinga Nagar, Bhubaneswar, India; ^2^​ Scientist-E, RMRC (ICMR), Bhubaneswar, Odisha, India

**Keywords:** broth microdilution, colistin broth disc elution, colistin resistance, Vitek 2

## Abstract

**Introduction.:**

The increasing antibiotic resistance like the advent of carbapenem resistant Enterobactarales (CRE), Carbapenem Resistant *Acinetobacter baumanii* (CRAB), and Carbapenem Resistant *Pseudomonas aeruginosa* (CRPA) has led to to the use of toxic and older drugs like colistin for these organisms. But worldwide there is an increase in resistance even to colistin mediated both by chromosomes and plasmids. This necessitates accurate detection of resistance. This is impeded by the unavailability of a user-friendly phenotypic methods for use in routine clinical microbiology practice. The present study attempts to evaluate two different methods – colistin broth disc elution and MIC detection by Vitek two in comparison to CLSI approved broth microdilution (BMD) for colistin for Enterobactarales, *Pseudomonas aeruginosa *, and *Acinetobacter baumanii* clinical isolates.

**Methods.:**

Colistin susceptibility of 6013 carbapenem resistant isolates was determined by BMD, Colistin Broth Disc Elution (CBDE), and Vitek two methods and was interpreted as per CLSI guidelines. The MIC results of CBDE, Vitek two were compared with that of BMD and essential agreement (EA), categorical agreement (CA), sensitivity, specificity, very major error (VME), major error (ME) and Cohen’s kappa (CK) was calculated. The presence of any plasmid-mediated colistin resistance (mcr-1, 2, 3, 4 and 5) was evaluated in all colistin-resistant isolates by conventional polymerase chain reaction.

**Results.:**

Colistin resistance was found in 778 (12.9 %) strains among the carbapenem resistant isolates. *Klebsiella pneumoniae* had the highest (18.9 %) colistin resistance by the BMD method. MIC of Vitek two had sensitivity ranging from 78.2–84.8% and specificity of >92 %. There were 171 VMEs and 323 MEs by Vitek two method, much more than CLSI acceptable range. The highest percentage of errors was committed for *Acinetobacter baumanii* (27.8 % of VME and 7.9 % ME). On the other hand, the CBDE method performed well with EA, CA, VME and ME within acceptable range for all the organisms. The sensitivity of the CBDE method compared to gold standard BMD varied from 97.5–98.8 % for different strains with a specificity of more than 97.6 %. None of the isolated colistin resistant organisms harboured *mcr* plasmids.

**Conclusion.:**

As BMD has many technical complexities, CBDE is the best viable alternative available for countries like India. A sensitive MIC reported by Vitek two needs to be carefully considered due high propensity for VMEs particularly for *Klebsiella* spp.

## Data Summary

The authors confirm all supporting data, code and protocols have been provided within the article or through supplementary data files.

## Introduction

In 2019, 4.95 million deaths were attributed to bacterial anti-microbial resistance (AMR) which is projected to rise further in the future [1]. Increasing drug resistance among Gram-negative bacilli (GNB) worldwide has reduced the essential antibiotic arsenal [2]. There is rapid expansion of carbapenemase producing Enterobacterales (CRE), *Acinetobacter baumanii* (CRAB), *Pseudomonas aeruginosa* (CRPA) and carbapenem heteroresistance in strains like *Enterobacter cloacae *. These bacteria are often resistant to other available classes of antibiotics. The high prevalence of metallo beta-lactamases presides against the use of less toxic alternatives like ceftazidime avibactam in India. Newer beta lactamase inhibitors and ceftiderocol are costly and not available in India. To counter this issue, older antibiotics like colistin were ameliorated and revived as a last resort toxic alternative. But now there are reports of colistin resistance mediated by chromosomal mutations and plasmid transfer [3]. In India, colistin resistant rates have been reported to be as high as 40 % in carbapenem resistant *K. pneumoniae* and 10 % in carbapenem resistant *E. coli* [4]. These rates seem to be increasing by the day with the added fear of plasmid (*mcr*) mediated resistance.

The cationic nature of the colistin molecule impedes the suitability of disc diffusion as well as E-test methods for susceptibility testing. Broth microdilution (BMD) performed with colistin sulphate in cation-adjusted Mueller-Hinton broth in polystyrene trays without any other added surfactant is the recommended method for testing [5]. But this is often labour intensive in diagnostic settings. Another method which was put forth by Simner *et al*. [6], was the colistin broth disc elution (CBDE) method, which is easier to perform in a clinical microbiology lab. It is accepted by CLSI for Enterobactarales and *Pseudomonas* spp. but is not yet approved for *Acinetobacter* spp. [5]. This method challenges a known concentration of test organism with a graded concentration of colistin (1, 2, 4 µg ml^−1^) achieved by eluting 10 µg colistin discs (1, 2, 4 respectively) into 10 ml of Cation Adjusted Mueller Hinton broth. Automated methods like Vitek two (bioMérieux, France) are the most convenient methods and often serve as a backbone of labs. Thus its use to perform colistin susceptibility needs to be evaluated.

This present study focused on evaluating the performance of the Vitek two automated system (bioMérieux, France) and CBDE method for organisms of Enterobacterale family, *Acinetobacter baumanii* and *Pseudomonas aeruginosa* from ICUs, the harbinger places of colistin resistant bugs. Additionally the *mcr* 1–5 gene was detected in the colistin resistant isolates in a view to assess the performance of the testing methods in phenotypic detection of *mcr* based resistance.

## Methods

### Study settings

This study was conducted prospectively in samples received at the Central Laboratory of the Institute of Medical Science and SUM Hospital, Odisha, India from June 2020–June 2022.

### Bacterial strains

During this study period, 6013 significant, non-repetitive carbapenem resistant Gram-negative bacilli were collected from various clinical samples of patients from the Intensive Care Unit (ICU). The samples included endotracheal aspirates, bronchoalveolar lavage fluid, blood, pus, wound swabs, tissue biopsy, urine, and sterile body fluids which were collected at bedside by trained personnel following the standard operating procedures. The samples were cultured on blood agar, chocolate agar, maconkey agar and colonies obtained on them were subjected to Gram-staining, motility, catalase and oxidase tests as per routine microbiological techniques. Further identification and sensitivity pattern of these bacteria was performed by Vitek two automated system (bioMérieux, France). Strains with MIC of imipenem >1 µg ml^−1^, doripenem >1 µg ml^−1^ or, meropenem >1 µg ml^−1^ or ertapenem >0.5 µg ml^−1^ were considered as carbapenem resistant. On account of being intrinsic resistant *Serratia marcescens *, *Proteus mirabilis, Morganella morganii, Proteus vulgaris, Providencia stuartii* and *Providencia rettgeri* were excluded from the Enterobactarales in the study. Susceptibility testing for colistin was performed for all the isolates by three different methods – CBDE, BMD and Vitek two automated system.

### Detection of colistin resistance by Vitek two

Manufacturer’s instruction was used for determining colistin Minimum Inhibitory Concentrations (MIC) by commercial Vitek two AST system by using AST-N280 and AST-N281 cards. (bioMérieux, France).

### Broth microdilution method for detection of colistin resistance

Broth microdilution with MIC range: 0.25–16 mg l^−1^ was performed based on ISO-20776–1 protocol [7] for all the 6013 isolates. Colistin sulphate powder (PCT1142-HiMedia Labs) was diluted with sterile distilled water as per the manufacturers' instructions. Then 25 µl each of the two-fold serially diluted colistin solutions was put into the different wells of flat bottomed 96 well microtitre plate (Tarson) and in each row. An overnight 37 °C incubated agar plate of the test organism was used and its colonies were directly suspended in normal saline to obtain turbidity standardized to 0.5 McFarland (1.5×10^8^ c.f.u. ml^−1^) which was further diluted 1 in 75 to obtain the standardized inoculums (2×10^6^). From this, 25 µl of the inoculum was added to each well within 15 min of its preparation and 50 µl of CAMHB media (Himedia Labs M1657) to arrive at the final inoculum in each well (5×10^5^ c.f.u. ml^−1^). The inoculated microtitre plates were incubated at 35±2 °C for 16–18 h following which MIC was recorded by unaided eye. Each row contained a growth control to check the viability of the organism and a negative control for checking the broth media for any contamination.

### Colistin broth disc elution method

Four tubes of Cation adjusted Muller Hinton Broth were taken for each of 6013 isolates and labelled as 0 (for control), 1, 2 and 4 µg ml^−1^ and to them one, two and four colistin sulphate 10 µg discs (Himedia Labs) were added respectively. These were vortexed and placed for 30 mins, following which 50 µl of the inoculums adjusted to 0.5 Mc Farland was added to each of these tubes to attain a final concentration of approximately 7.5×10^5^ c.f.u. ml^−1^. These tubes were further vortexed and incubated at 35 °C in ambient air for 16–20 h. A purity check was performed from inoculums and MIC was read as the lowest concentration of the antibiotic that completely inhibited the growth of the isolate.

### Interpretation

CLSI MIC breakpoints of >2 mg l^−1^ for resistance and ≤2 mg l^−1^ for intermediate were utilized in the study [5].

### DNA extraction and polymerase chain reaction for mobile colistin resistance (*mcr*) gene

Genomic DNA extraction performed for all 6013 samples was done using the boiling method [8]. The extracted DNA was standardized using a nanodot to 40 ng µl^−1^. Conventional PCR was done for all these samples using a QIAGEN Taq PCR master mix kit and primers from Eurofins for *mcr-1, mcr-2, mcr-3, mcr-4, mcr-5* genes (Table 1). Gel electrophoresis was done to observe the amplification of the desired gene with 1.5 % agarose gel.

**Table 1.** List of primers used for the detection of plasmid mediated colistin resistance

Gene	Primer	Amplicon size	Melting point	Reference
mcr-1	F-CGGTCAGTCCGTTTGTTC	309 bp	58 °C	Ahmed *et al*. [38]
	R-CTTGGTCGGTCTGTAGGG			
mcr-2	F-TGGTACAGCCCCTTTATT	1747 bp	49 °C	Yang *et al*. [39]
	R-GCTTGAGATTGGGTTATGA			
mcr-3	F-TTGGCACTGTATTTTGCATTT	542 bp	54 °C	Roer *et al*. [40]
	R-TTAACGAAATTGGCTGGAACA			
mcr-4	F-ATTGGGATAGTCGCCTTTTT	487 bp	52 °C	Roer *et al*. [40]
	R-TTACAGCCAGAATCATTATCA			
mcr-5	F-ATGCGGTTGTCTGCATTTATC	1541 bp	50 °C	Borowiak *et al*. [41]
	R-TCATTGTGGTTGTCCTTTTCTG			

### Quality control

Manufacturer’s instructions were followed for quality control testing of the Vitek two instrument. *E. coli* NCTC 13846 (colistin MIC- 4 µg ml^−1^) and *E. coli* ATCC 25922 were used in each batch of BMD and CBDE tests as quality control organisms. For each new bottle of procured cation adjusted muller hinton broth *Pseudomonas aeruginosa* ATCC 27853 with imipenem (MIC-1–4 µg ml^−1^) was used for assessing the media quality.

### Statistical analysis

All three methods were conducted in parallel and results obtained by Vitek two and CBDE were compared with the gold standard BMD method. Essential agreement (EA) was the MIC of the test method that was varying by one log2 dilution of reference method expressed in percentage. Categorical Agreement was the similarity of categories (resistant or sensitive) by test and reference BMD method expressed in percentages. Major Error (ME) percentage was identified as a colistin-susceptible isolate (by BMD) that was misinterpreted as a colistin-resistant strain (by test method) divided by true susceptible (by BMD) isolates. Similarly, Very Major Error (VME) percentage was calculated as the number of colistin susceptible strains by the test method (CBDE and Vitek two) which is a colistin-resistant strain by BMD divided by true resistant (by BMD) isolates. Acceptable agreement for was defined as CA≥90 %, EA ≥90 %, VME≤1.5 % and ME ≤3 % as per CLSI guidelines [5]. Sensitivity, measuring true positive and specificity measuring true negative portion [9] were calculated. Positive Predictive Value (PPV) is the strains giving resistant test results, which are true resistant (by BMD). Negative Predictive Value (NPV) is the isolates giving sensitive test results, which are true sensitive (by BMD) [9]. Test reliability was calculated using Cohen’s Kappa (CK) statistics that measure inter-rater agreement was calculated and interpreted as below (Table 2) [10].

**Table 2.** Cohen’s Kappa value and their interpretation

Kappa value	Interpretation of reliability
0–0.2	None
0.21–0.39	Minimal
0.4–0.59	Weak
0.6–0.79	Moderate
0.8–0.9	Strong
>0.9	Almost perfect

### Ethical approval

This study was conducted following approval from the Institute’s ethics committee via- no. IEC/IMS.SH/SOA/2O22/290. All the samples were received during the purpose of diagnosis in the laboratory. The protocols followed for the tests were within patient care standards. No patient data was disclosed in any form and no diagnostic or therapeutic activity was hampered in this study.

## Results

In the present study of the total 6013 carbapenem resistant isolates, *E. coli* (2255; 37.5 %) were the most common species followed by *Klebsiella pneumoniae* (1690; 28.1 %) and *Enterobacter cloacae* (635; 10.6 %). Among non-Enterobactarales there were 731 (12.2 %) *Pseudomonas aeruginosa* and 702 (11.67 %) *Acinetobacter baumannii* strains. Samples from which the strains were isolated were endotracheal aspirates (13 %), bronchoalveolar lavage fluid (10 %), blood (23 %), pus (15 %), wound swabs (13 %), tissue biopsy (6 %), urine (9 %), pleural fluid (5 %), and ascitic fluid, knee aspirate, and synovial fluid (3 %). Among these 778; 12.9 % of strains were colistin resistant by the gold standard BMD test. When different species were considered, *Klebsiella pneumoniae* showed the highest percentage of colistin resistance 319 (18.9 %) while in *Pseudomonas aeruginosa* 117 (16 %); *Acinetobacter baumanii* 97 (13.8 %); *E. cloacae* 66 (10.4 %) and *E. coli* 179 (7.9 %) strains were colistin resistant.

When the results of Vitek two were compared to MICs obtained by BMD the sensitivity ranged from 78.2–84.8% and specificity was more than 92 % in case of all the organisms (Table 3). Thus Vitek two has a chance to report a large number of isolates as false susceptible to colistin while false resistance may not be as high. The PPV also varied from 58.4–78.37 %, while the NPV was between 93.4 and 98.48 %. EA of the Vitek two method varied between 71 % in *Klebsiella* spp. to 88 % in the case of *E. coli *, which is not acceptable as per CLSI standards of EA >90 %. Vitek two MIC for colistin also did not meet the CA standards for *P. aeruginosa* and *Klebsiella pnuemoniae* (Table 3). There were 171 VMEs and 323 MEs by the Vitek two method of which the highest percentage were committed for *Acinetobacter baumanii* (27.8 % of VME and 7.9 % ME).

**Table 3.** Performance of CBDE and Vitek two in comparison to reference BMD method for detecting colistin resistance

Organism	Method	R	I	MIC_50_	MIC_90_	VME (%)	ME (%)	EA (%)	CA (%)	SEN (%)	SPE (%)	PPV (%)	NPV (%)	Cohen’s Kappa and interpretation
* E. coli * *n=*2255	BMD	179	2076	2	4									
	VITEK2	211	2044			32 (17.8)	127 (6.11)	257 (88.6)	201 (91.1)	84.8	94.23	58.4	98.48	0.03- None
	CBDE	197	2058			2 (1.1)	54 (2.6)	33 (98.5)	98 (95.7)	98.9	97.46	76.8	99.40	0.83- Strong
* K. pneumoniae * *n=*1690	BMD	319	1371	2	16									
	VITEK2	457	1233			77 (24.1)	88 (6.41)	487 (71.2)	347 (79.5)	80.5	94	78.4	93.40	0.04- None
	CBDE	388	1302			6 (1.9)	31 (2.26)	88 (94.7)	41 (97.6)	98.2	97.78	91.1	99.56	0.606- Moderate
* A. baumannii * *n=*702	BMD	97	605	2	8									
	VITEK2	124	578			27 (27.8)	48 (7.9)	88 (87.5)	54 (92.3)	78.2	92.64	66.8	95.72	0.027- None
	CBDE	109	593			2 (2.1)	5 (0.82)	31 (95.6)	12 (98.2)	97.9	99.18	95.1	99.67	0.87- Strong
* P. aeruginosa * *n=*731	BMD	117	616	2	16									
	VITEK2	138	593			21 (17.8)	34 (5.51)	101 (86.2)	121 (83.4)	84.8	94.76	77.5	96.73	0.13- None
	CBDE	126	605			3 (2.6)	12 (1.94)	55 (92.5)	30 (95.9)	97.5	98.08	90.6	99.51	0.79- Moderate
* E. cloacae * *n=*635	BMD	66	569	1	16									
	VITEK2	98	537			14 (21.1)	26 (4.5)	79 (87.5)	39 (93.9)	82.5	95.63	73.3	97.59	0.22- None
	CBDE	89	554			1 (1.51)	5 (0.87)	27 (95.7)	22 (96.5)	98.5	99.12	92.9	99.82	0.83- Strong
Total *N*=6013		778												

The sensitivity of the CBDE method compared to gold standard BMD varied from 97.5–98.8 % for different species with a specificity of more than 97.6 %. The PPV ranged from 76.82–95.09 %, whereas the NPV was recorded as more than 99.4 %. The EA of the CBDE method ranged within acceptable limits for all the organisms. EA levels were highest in *E. coli* by both methods and lowest in *Klebsiella pnuemoniae* (71.18 %) for Vitek two and *Pseudomonas aeruginosa* (92.47 %) for CBDE. CA was met for the CBDE method for all the bacteria (Table 3). There were 14 VMEs and 107 MEs by the CBDE method.

**Fig. 1.**
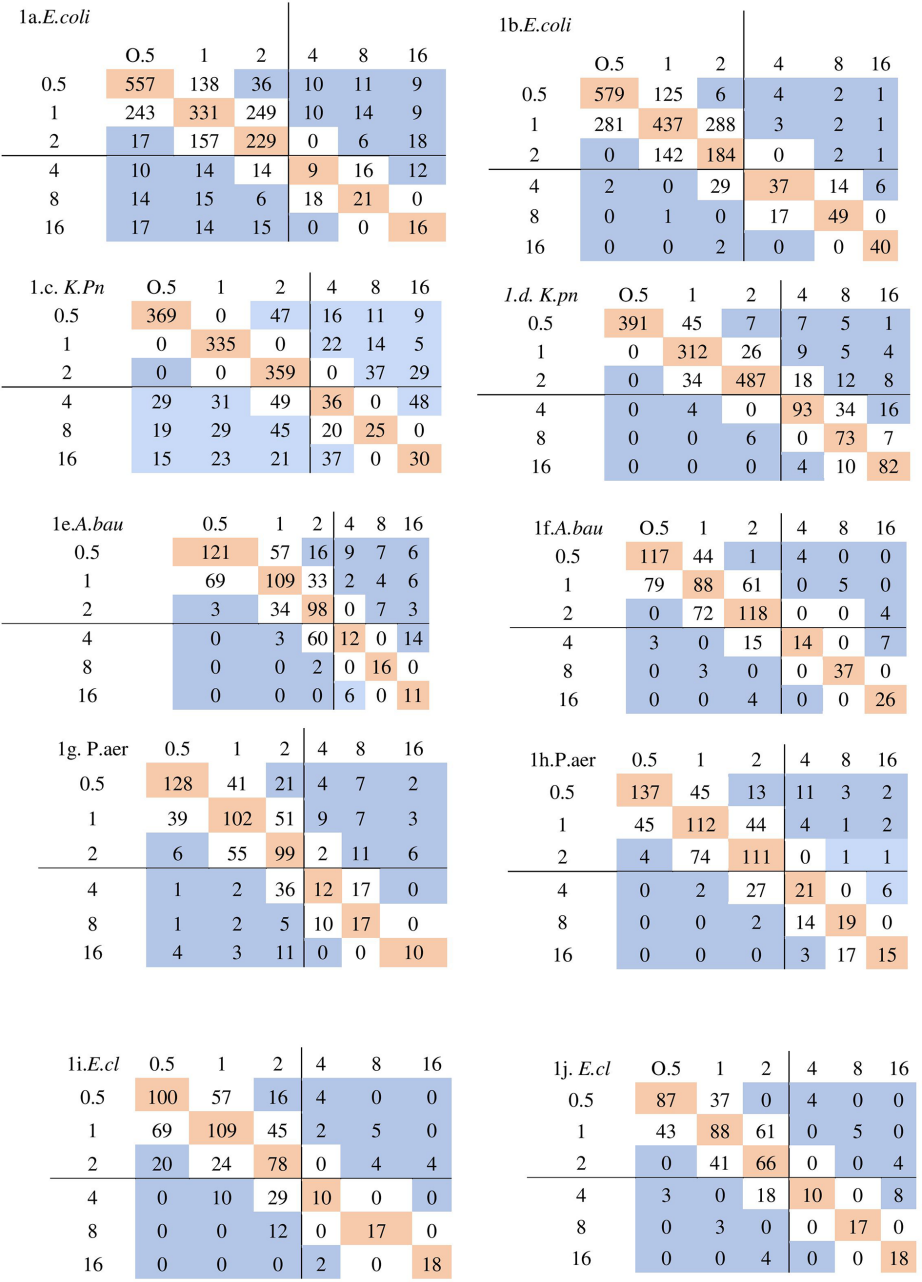
Scatter diagram obtained by plotting MIC obtained by reference BMD method (x-axis) with MIC by test method i.e. Vitek two/CBDE (y-axis). Bold lines represent the breakpoint MICs in micrograms per millilitre. The pink colour boxes are the isolates showing perfect agreement. The essential agreement is highlighted by both pink and white colour boxes. 1 a. MIC of BMD vs Vitek2 for *E. coli (Escherichia coli),* 1 b. MIC of BMD vs CBDE for *E. coli* 1 c. MIC of BMD vs Vitek2 for *K.pn (Klebsiella pneumoniae),* 1 d. MIC of BMD vs CBDE for *K.pn* 1e. MIC of BMD vs Vitek2 for *A.bau (Acinetobacter baumanii),* 1 f. MIC of BMD vs CBDE for *A.bau* 1 g. MIC of BMD vs Vitek2 for P.aer (*Pseudomonas aeruginosa),*1h. MIC of BMD vs CBDE for *P.aer* 1i. MIC of BMD vs Vitek2 for *E.cl* (*Enterobacter cloacae),* 1 j. MIC of BMD vs CBDE for *E.cl*.

The performance of CBDE and Vitek two in reference to BMD is shown in Fig. 1 determining the MICs of colistin. In general, the MIC values by BMD for all of the isolates examined were greater than those obtained by CBDE and Vitek two. Vitek two’s correlation with reference MICs was weak, and a 45 degree correlation could not be achieved. Vitek two has a propensity of underestimating MICs for resistant isolates. Cohen’s Kappa value suggests the reliability of the tests was strong for CBDE for all the isolates except for that of *Klebsiella pneumoniae* where it was moderate. None of the samples harboured *mcr* genes.

## Discussion

Colistin is the last line of antibiotic for carbapenem-resistant Gram-negative bacteria like *K. pneumoniae, E. coli, A. baumanii* and *P. aeruginosa* [11]. Reliable identification and reporting of its susceptibility pattern is of utmost importance for administering colistin in healthcare settings. In our study, colistin resistance among CRE was 12.9 % with standard BMD technique which is comparable to findings presented in other studies [2, 12]. Studies testing colistin susceptibility using BMD in Greece, Italy, India, Egypt, Germany, and Poland found resistance rates of 95.1, 13.5, 32.4, 45.1, and 17 %, respectively [13–18]. In the present study, colistin resistance was more in *K. pneumoniae* (18.9 %) than *E. coli* (7.9 %) as per observations published in previous literature [2, 19, 20]. Among *Pseudomonas aeruginosa* isolates 16 % were colistin resistant. In the study by Pawar *et al*. [21], 51.8 % of colistin resistant were *Pseudomonas aeruginosa* while in the study by Jain *et al*. [22], 18 % of *Pseudomonas* spp. were colistin resistant.

MIC50/90 of *E. coli* (2/4 µg ml^−1^) by BMD method was lower than MIC50/90 of other bacilli (2/16 µg ml^−1^). The presence of capsular polysaccharide causes the higher MIC range of colistin in *K. pneumoniae* [23]. Worldwide there is a trend of increasing colistin MIC levels [24, 25] thus necessitating a proper colistin susceptibility testing method.

The susceptibility testing for colistin is challenging due to poor diffusion of the drug in agar, cationic property of colistin and heteroresistance in MDR organisms [26]. There is no agreement between colistin susceptibility testing methodologies [27]. BMD, the gold standard method adopted is time- and resource-consuming [18]. The techniques frequently used in routine laboratories is disc diffusion and E-test, but have been shown to be inefficient for colistin [28].

Several laboratories depend on automated methods like the automated method Vitek two system. A recent study [18] found solid agreement between BD Phoenix automated system and BMD for colistin susceptibility testing. Thus it is critical to investigate the performance of Vitek two in this direction which we attempted with a large sample size including both colistin sensitive and resistant strains. In the present study when compared to the gold standard; the automated Vitek two approach had unacceptable EA for all the organisms. CA was unacceptable for *Klebsiella pneumoniae* and *Pseudomonas aeruginosa *. Previous studies have also shown ambiguous conclusions for Enterobacterales, with some showing a high rate of EA and CA without any VME [14, 29]. Both VME and ME of Vitek two were not within acceptable standards in the present study as in other studies [30, 31]. In other studies VME is as high as 36 % [12, 16, 32]. This high VME and ME may be due to the use of plastics in this automated system which is shown to absorb the drug to its surface. All the phenotypic methods performed better against *E. coli* in comparison to *K. pneumoniae *, as in previous studies [12].

All strains fulfilled the EA and CA of >90 % when tested with the CBDE method in the present study. A similar result of 88.9 and 97.8 % CA was noted in studies by Shams *et al*. [18] and Humphries *et al*. [33] was also obtained in previous studies. Disc elution is easier to perform, interpret and has higher affordability in comparison to other methods and is a CLSI endorsed test for Enterobactarales and *Pseudomonas aeruginosa *. Simner *et al.* [6] found 98 % CA, 99 % EA and no errors with this method. Koyuncu *et al.* found 99 % CA with 0.5 % very major errors [34]. In our study, the EA and CA were acceptable with 14 VMEs and 97 MEs by CBDE. The dissimilarity in results may be caused due to discs produced by different manufacturers. A MIC of two may warrant further testing, particularly for *Klebsiella pneumoniae *. We found a PPV of >90 % of all bacteria except *E. coli* (76.8 %) which is comparable to that of Shams *et al.* [18] who had a PPV of 95.56 % for the CBDE method. But we had a much better NPV for the CBDE test (>93 %) than that of Shams *et al.* (80 %) [18]. Although still not endorsed by CLSI, we found acceptable EA, CA and VMEs while using the CBDE method for *Acinetobacter baumanii* as well.

Plasmid mediated (*mcr*) colistin resistance was first detected in 2015 in food industries in China and since then has been reported in various other countries [35]. But in our study no *mcr* 1–5 mediated resistance was detected similar to other studies from India [1, 36]. Simner *et al.* recommended that *mcr* related colistin resistance may be further confirmed by BMD [6]. As *mcr* is not yet prevalent in clinical strains in India CBDE may be adopted as an easy colistin susceptibility testing method. One of the automated platforms BD Phoenix has been proposed as a reliable tool for the detection of *mcr* mediated colistin resistance [37], but Vitek two results in view of *mcr* genes need to be evaluated further.

The isolates were taken from one centre and susceptible isolates were much more than resistant strains. Due to a large number of isolates and a lack of adequate manpower triplicate testing of the isolates were not performed. There were no *mcr* positive strains in the present work and no positive control was run. Sequencing studies of the colistin resistant isolates were not undergone. These are the limitations of the present study. Heteroresistance in colistin and the clinical importance of *mcr* mediated resistance should be the topics for further research.

The panel of antibiotics that can be administered in case of carbapenem resistant bacteria is limited to a few like ceftazidime avibactam, ceftalozane tazobactam, tigecycline and colistin. As India has a predominance of *ndm-1* metallo beta lacatamases, colistin often is the only drug at hand. Thus every laboratory should perform appropriate colistin susceptibility testing. As BMD has many technical complexities, CBDE is the best viable alternative available for countries like India. We also found good agreement between CBDE and BMD in susceptibility testing of *Acinetobacter baumanii*. A sensitive MIC reported by Vitek two needs to be carefully considered due high propensity for VMEs particularly for *Klebsiella* spp.

## Supplementary Data

Supplementary material 1
